# Curcumin-Galactomannoside Complex inhibits the Proliferation of Human Cervical Cancer Cells: Possible Role in Cell Cycle Arrest and Apoptosis

**DOI:** 10.31557/APJCP.2021.22.6.1713

**Published:** 2021-06

**Authors:** Ratheesh M, Svenia P Jose, Krishnakumar IM, Sandya S, Sangeeth Saji, Sheethal S

**Affiliations:** 1 *Department of Biochemistry, St. Thomas College, Pala, Kottayam, Kerala, India. *; 2 *R & D Center, Akay Natural Ingredients Pvt.Ltd, Cochin, India. *; 3 *Inorganic and Physical Chemistry, Indian Institute of Science, Bangalore, Karnataka, India. *

**Keywords:** Apoptosis, Bcl-2, caspase-8, cervical cancer, ROS

## Abstract

**Background:**

Cervical cancer is the most common cancer and has the highest morbidity rate of gynaecological malignancies in women worldwide. So, the development of effective anti-cancer agents to treat this condition is vital. Considering the recent interest in free (unconjugated) curcuminoids delivery, the present study investigated the efficacy of a novel food-grade, free-curcuminoids (curcumin-galactomannoside complex; CGM) on cervical cancer cells (HeLa) of human origin. In this study, we examined the anticancer potential of CGM as well as its effects on the cell cycle and the apoptosis of HeLa cancer cell.

**Methods:**

Determination of anti-proliferative and apoptosis validation of CGM on HeLa cells was performed by 3-(4,5-Dimethylthiazol-2-yl)-2, 5,-diphenyltetrazolium bromide (MTT), acridine orange/propidium iodide and annexin-V-fluorescein isothiocyanate assays. Measurement of Reactive Oxygen Species (ROS) production, Caspase activities and protein expression experiments were performed to investigate the potential mechanisms of action in the apoptotic process.

**Results:**

The cytotoxic assays revealed that the CGM showed inhibition of cell survival and exhibited high cytotoxic activity against HeLa cells at 25 μg/mL. Further studies on morphological changes were done in CGM-treated cervical cancer cells contributing to apoptosis. Flow cytometry analysis with Annexin V-FITC and PI staining precisely indicated that CGM induced apoptosis in HeLa cell lines at 25 μg/mL. By the supplementation of CGM showed an increase in Bax and cleaved caspase-8 protein in HeLa cells after 48 h exposure.

**Conclusion:**

The evidence obtained from this study suggests that CGM is a potent and promising natural formulation against cervical cancer cells via induction of apoptosis through ROS mediated mitochondrial damage in HeLa cells. Hence, CGM could be further explored as a potential lead in treating cancer.

## Introduction

Cancer is a major life-threatening disease and one of the foremost stimuli of death all over the world. Among diverse cancers, Cervical Cancer (CC) is the one that stands in the third position worldwide and the fourth leading cause of death from gynaecologic malignancy. The recent disclosure from World Health Organization (WHO) showed that the highest incidence of the disease is estimated to occur in the developing countries including India and is also the second most prevalent and largest killer among women, surpassing breast cancer. Current estimates indicate that every year, over 529,000 of total new cases and approximately 275,000 mortalities are caused by CC in the world, which accounts for 87% of all cancer mortalities in females (Arbyn et al., 2011).

The greater risk factor for the development of CC is a consequence of long-term infection with high-risk oncogenic Human Papilloma Virus (HPV) type encoding two oncoproteins, E6 and E7 (Ledwaba et al., 2004;Walboomers et al.,1999), which are important for malignant transformation of CC (Zur Hausen, 1994). Although the women of all ages, especially from 30 to 55 are more susceptible to CC (Agarwal et al., 2011) due to HPV infection and few other factors such as smoking, working stress, etc. During the HPV infection, its genome gets integrated into the host body, which further leads to dysregulation of the cellular processes including cell proliferation, alteration of growth and differentiation factors, DNA synthesis, and oxidative stress and eventually leads to CC formation. The foremost potent defence mechanism against cancer cells is apoptosis, which mainly regulates tissue homeostasis through the elimination of potentially deleterious cells. Oxidative stress is an imbalanced redox status of the cell which leads to the generation of reactive oxygen species and thereby occurred a detrimental condition like cell death (Jaudan et al., 2018). Apoptosis is the mechanism of programmed cell death and many in-vitro studies demonstrated that the molecular events of apoptosis were accompanied by the regulation of Bcl-2, Bax, activation of caspases, inhibition of cell proliferation through G0/G1 cell cycle arrest, and induced apoptosis via mitochondrial apoptotic pathway in human cervical cancer cells (Xu et al., 2017).

Nowadays, the recurrence rate of CC is still high in most countries, on the other side most of the anti-cancer drugs, radical surgery and radioactive rays suppress DNA damage or duplication and thereby it kills the cancer cells (Wang and Qiao 2015). Although, they might have concerns with normal cells in giving rise to several undesirable side-effects, such as bone marrow function inhibition, nausea, vomiting, alopecia, toxicity and Multiple drug resistance (MDR) (Sak,2012; Baskar et al.,2014). Obviously, CC remains a common cause of tumor-death in women in developing countries due to lack of access to screening (Pap test) or vaccines against HPVs. The conventional treatment strategies used in CC are surgery, radiation and chemotherapy. However, all of these advanced treatment approaches may lead to several side-effects as well as acquired chemo-resistance inside the body. Nevertheless, each of these conventional treatments has its own severe side effects. Moreover, the increase in the death rate due to CC and the adverse effects of anti-cancer drugs available in the market are the main challenges to the researchers to come across new, safety and more effective drug with fewer side-effects. 

From the above-mentioned drawbacks, now the researchers are more concerned about the novel therapeutic approach to alternative medicines which might be capable of curing cancer. Therefore, a new therapeutic level for the treatment of cervical cancer is essential. Despite advances in the treatments of CC, the abuse of natural constituents is one of the most flourishing methods to blemish a new channel against CC. For instance, the uptake of drugs may be promoted by the improved permeability as well as the releasing effect which may enhance the drug accumulation in the target site in cancer cells; hereby it down regulates the progression and aggressive stage of cancer. In addition, the time consuming and controlled release of drug is more helpful to improve the efficiency and safety in the treatment stage. Also, most of the available clinical studies are focused on bioavailability, pharmacokinetics and metabolism of novel formulations. For the treatment of many diseases, medicinal plants or their active components have great acceptance, especially the components which are derived from food are widely used due to the side effects of other synthetic medicines (Sharma et al., 1994). 

Curcumin (diferuloylmethane) is one such compound and it is used as both flavouring and colouring agent in Asia (Cooper et al., 1994). Various studies have been revealed that curcumin prompt drastic alterations in cell metabolism, cell cycle, and carcinogenicity of tumour proteins in HeLa cells (Sarkhosh et al., 2018). Due to the low solubility and poor bioavailability, naturally available curcumin cannot attain its optimum therapeutic effects. 

So that a novel enhanced bioavailable formulation of curcumin as ‘curcumagalactomannosides’, using a unique composition of soluble dietary fibre (galactomannans) and proteins isolated from the fenugreek seeds (hereinafter referred to as ‘CGM’, patented and registered formulation as CurQfen^®^) was developed (Krishnakumar et al., 2012).The current findings from Roy and Mukherjee (2014) explains the reversal of resistance to cisplatin by adding curcumin in cisplatin-resistance cervical cancer cell lines. In addition, Basu et al., (2013) demonstrated that the positive therapeutic response for HPV infection in 289 HPV positive women with the use of curcumin containing polyherbal vaginal cream as well as curcumin containing vaginal capsules for period of 1 month. Curcumin independently has shown to inhibit IL-6 activity and the NF-kB pathway in cancer cell lines (Panahi et al., 2014). The present study aimed to determine the anti-proliferative effect of bio-available formulation of curcumin galactomannoside complex (CGM) through the induction of apoptosis in cervical cancer cells.

## Materials and Methods

*Chemicals*

MTT, acridine orange (AO), ethidium bromide (EB) and propidium iodide (PI) were purchased from Sigma-Aldrich. Cell apoptosis and cell cycle analysis kits were purchased from BD Pharmingen™, Cat No: 559763, San Diego, CA, USA. All other chemicals and reagents used in the present study were of the analytical grade.

*Cell treatments*

The cancer cell line used in this research was the HeLa cervical cell line. The cells were obtained from the National Centre for cell sciences, Pune, India. It was maintained in Dulbecco’s Modified Eagle Medium supplemented with 10% fetal bovine serum, 100 µg/ml streptomycin and 100 U/ml penicillin G in an incubator at 37°C with 5% CO_2_. 

*Assessment of cell viability by MTT assay*

The cytotoxic effect of CGM was assessed by using MTT assay (Mosmann, 1983). In brief, cells were seeded at a concentration of 1×10^4^ cells/ml in a 96-well plate. Cells were treated with various concentrations of CGM (5μg/ml, 12.5μg/ml, 25μg/ml, 50μg/ml and 100μg/ml) for 24 h. Then 20μl MTT (3-(4, 5- dimethylthiazol- 2-yl)-2,5-Diphenyl tetrazolium bromide) was added to each well and incubated at 370C for 4 hrs. Then, the medium was removed and formazan was dissolved in 200µl/well DMSO and mixed several times by pipetting up and down. The absorbance was read at 570nm using ELISA reader. The results were expressed as viability (%) as compared to control-treated cells. 

*Neutral Red Uptake (NRU) Assay*

The in-vitro cytotoxicity activity was performed using the NRU assay using the procedure (Siddiqui et al., 2010). Here, the HeLa cells were treated with 5μg/ml, 12.5μg/ml, 25μg/ml, 50μg/ml and 100μg/ml CGM for 24 h. Then, cells were washed with PBS twice and incubated further in 50 µg/ml of neutral red containing medium for 3 h. Then cells were washed off with a solution (1% CaCl2 and 0.5% formaldehyde). The dye was extracted in a mixture of 1% acetic acid and 50% ethanol. The absorbance was measured at 550 nm using ELISA reader. The results were expressed as a percentage as compared to control-treated cells. 

*Morphological Analysis*

The observation of morphological changes of apoptotic cells was performed using a phase-contrast inverted microscope. Briefly, cells were incubated for 24 hours with various concentrations of CGM (5μg/ml, 12.5μg/ml, 25μg/ml, 50μg/ml and 100μg/ml) in a 60 mm diameter tissue culture dishes. The medium was discarded and cells were washed once with PBS. The changes in the morphology of the apoptotic cells were observed to determine the alterations in HeLa cells and the images of the cells were grabbed at 20x by using the phase-contrast inverted microscope (Olympus CKX 41, USA).

*Estimation of Reactive oxygen species (ROS) generation*

Reactive oxygen species (ROS) generation was assessed using a cell-permeable fluorescent signal 2’,7’-dichlorodihydrofluorescein diacetate (H2 DCFDA) as an indicator (Min et al., 2014; Wu et al., 2011). As described previously, H2 DCFDA is oxidized to a highly green-fluorescent 2070- dichlorofluorescein (DCF) by the generation of ROS. HeLa cell lines were treated witha concentration of 25µg/mL CGM for 24h. After 24 h incubation period, the cells were washed with cold PBS and incubated with 100 mmol/L H2 DCFDA for another 30 min at 37°C. DCF fluorescence intensity was measured using the fluorescence plate reader at excitation/emission of 488/525nm. The determinations were carried out thrice in triplicate, ensuring each time that the number of cells per treatment group was the same to ensure reproducibility. The values were expressed as % relative fluorescence and compared to the control. 

*Determination of apoptosis*

The cell apoptosis was performed using a commercial kit (BD Pharmingen™, Cat No: 559763, San Diego, CA, USA) following the manufacturer’s recommendations. HeLa cells were treated with Doxorubicin- 20µg/mL, CGM- 25µg/mL for 48 h, then the cells were trypsinized after incubation and 100 μL of cells in suspension was transferred to separate tubes. To the tubes added 100 μL of the Muse™ Annexin V and Dead Cell Reagent to each tube. The tubes were mixed thoroughly by pipetting up and down or vortexing at medium speed for 3 to 5 seconds followed by incubation for 20 minutes at room temperature in the dark. The cells were analyzed in a flow cytometer and using Muse flow cytometry software. Cells were gated again at untreated control cells and analyzed for apoptosis using Muse FCS 3.0 software.

*Cell cycle analysis*

Analysis of Cell cycle was performed using a commercial kit (BD Cycletest™ Plus, Cat No: 340242, San Diego, CA, USA) following the manufacturer’s recommendations. HeLa cells were treated with 25 μg/mL concentrations of CGM for 48 h, then harvested, and washed twice with buffer solution. The cells were incubated with trypsin solution for 10 min followed by trypsin inhibitor and RNase solution for 10 min. Finally, the cells were treated with propidium iodide (PI) staining on ice for 10 min. Data from 30,000 cells per sample were collected and analyzed on a flow cytometer (BD Accuri C6, MI, USA). The percentage of the cells in cycle phases was determined using MODFIT 3.0 verity software. The results were then compared with the untreated negative control cells.

*Western Blot Analysis*

The HeLa cells were plated and treated with CGM (25 μg/ml) for 48 h at 37 °C. Cell culture medium was then removed and cells were washed with PBS (pH, 7.3) and lysis (radio-immunoprecipitation assay) RIPA buffer, containing proteinase inhibitor phenylmethylsulfonyl fluoride (PMSF, 1 mM) and aprotinin (245 µg/ml). The lysate was centrifuged (11,400g) for 30 min at 4°C and the concentration of the protein was determined via BCA protein assay (Sigma Aldrich). An appropriate amount of proteins was boiled in a loading buffer, separated by SDS-PAGE, and transferred onto a nitrocellulose membrane. Membranes were blocked with milk (5% w/v) in TBST. Immunodetection was performed as described in the ECL kit protocol (Servicebio, China) and blots were incubated overnight at 4 °C with a specific antibody, washed with TBST, and incubated for another 30 min at room temperature with peroxidase-conjugated antibodies. Antibodies of Bax, caspase-8, cleaved caspase-8, Bcl-2, and β -actin was used as an internal control.

*Statistical analysis*

All the experiments were measured in triplicates. All data were presented as Mean ± standard deviation (SD) and the statistical significance was determined using the Student’t-test and the significance level was accepted at P < 0.05.

## Results

*Cytotoxicity Assessments of CGM and CM by MTT and NRU Assays*

**Figure 1:**
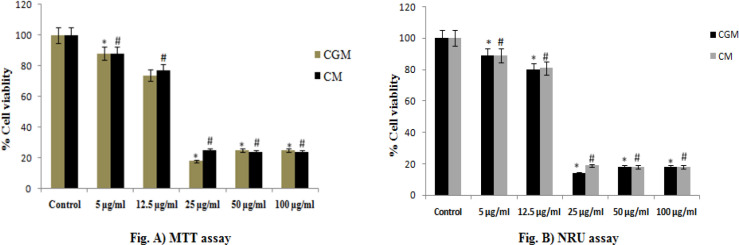
Cytotoxicity Assessments of CGM and CM by MTT and NRU Assays. Cytotoxicity assessment in HeLA cells following the exposure of various concentrations of CM and CGM for 24 h: (a) MTT assay; (b) neutral red uptake assay. The value represents mean ± SD of six values. *, # Statistical difference with control group at p ≤ 0.05

To evaluate the cytotoxicity of CGM and CM in HeLa cells, the effect of CGM and CM on the cell viability was evaluated using MTT assay. MTT is a yellow water-soluble tetrazolium salt. Succinate-dehydrogenase, a mitochondrial enzyme in living cells, cleaves the tetrazolium ring, converting the MTT to an insoluble purple formazan. Therefore, the amount of formazan produced is directly proportional to the number of viable cells. CGM and CM were screened for their cytotoxicity against HeLa cell lines at different concentrations. The key results obtained by MTT and NRU assays in HeLa cells exposed to 5 μg/ml to 100 μg/ml for 24 h are summarized in Figures 1(A) and 1(B). The results exhibited a concentration-dependent decrease in the viability of HeLa cells. The cell viability was recorded as 88% and 74% in CGM at 5 μg/ml and 12.5 μg/ml concentrations, respectively; however, the maximum decrease in cell viability was measured as 18% at 25 μg /ml of CGM as compared with CM. Like MTT assay, cell viability of HeLa cells exposed to CGM was also observed by NRU assay. The cell viability was recorded as 89% and 80% in CGM at 5 μg/ml and 12.5 μg/ml concentrations, respectively; however, the maximum decrease in cell viability was measured as 14% at 25 μg /ml of CGM as compared with CM. In this study, the cytotoxicity assessments were performed using two independent endpoints (MTT and NRU) assays (Nogueira et al., 2011). The MTT, a colorimetric assay is based on the mitochondrial dehydrogenase enzyme of viable cells (Mosmann, 1983), whereas NRU assay is based on the lysosomal integrity of viable cells (Borenfreund and Puerner, 1987). The cytotoxic responses of CGM indicated that a more bioavailable form of CGM could contribute in search of the alternative therapeutic agent in the CC treatment. Our results showed more than 60% of cell death even at 25 μg /ml of CGM. Therefore, at 25 µg/ml of CGM treatment was found to be the potent dose as compared with CM.

*Morphological observation of HeLa cells by CGM treatment*

**Figure 2:**
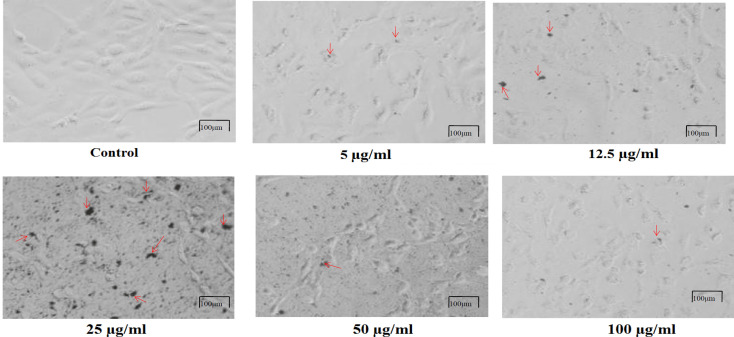
Morphological Observation of HeLa Cells by CGM Treatment. Images were taken using an inverted phase contrast microscope at 20x magnification. Control –Untreated HeLa cells. Different concentration of CGM at 5 µg/ml, 12.5 µg/ml, 25 µg/ml, 50 µg/ml, 100 µg/ml treated on HeLa cells. The morphology of cell changes and cell shrinkage were indicated by red arrows respectively. Scale bar 100μm

From the cytotoxic results, it was clear that the CGM is a potent and effective formulation against HeLa cells.In the present study, a significant morphological alteration was observed in HeLa cells treated with CGM at 5, 12.5, 25, 50 and 100 μg/ml for 24 h are shown in Figure 2. In control HeLa cells no significant changes were observed in the morphology and it displayed normal morphology, intact nuclear and cellular architecture and is typical of HeLa cells. However, the HeLa cells exposed to CGM lost their typical shape and cell adhesion capacity, shrunk and decreased cell density. From the morphological analysis, the cytotoxic effect of CGM may be due to the anti-neoplastic nature and their capability via numerous molecular mechanisms to induce cell death. 

*Evaluation of intracellular ROS level*

**Figure 3:**
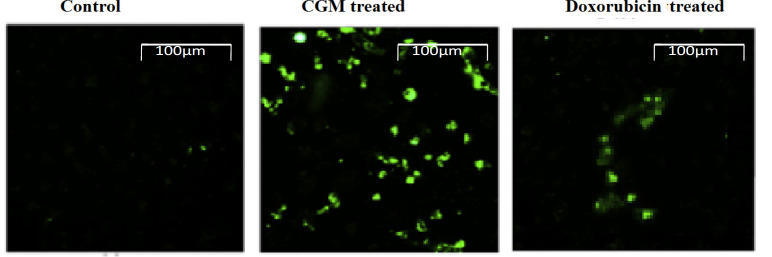
Evaluation of Intracellular ROS Level. Intracellular ROS generation by green fluorescence assay in HeLa cells exposed to CGM and standard drug doxorubicin for 24 h. Scale bar: 100μm

The production of intracellular ROS was measured via the conversion of non-fluorescent H2DCFDA to fluorescent DCFDA. Since the cytotoxicity of CGM was greater, a further investigation was done on CGM to reveal the mechanism for its cytotoxicity. From this point of view, the evaluation of the apoptotic activity through ROS generation of CGM against HeLa cell lines was carried out. The results showed that the CGM significantly increased the ROS production in HeLa cells (Figure 3). We found that exposure of HeLa cells with CGM dramatically enhanced the generation of intracellular ROS levels at 25 µg/ml concentration. Our results indicated that CGM induced intracellular ROS production as well as oxidative stress condition, thereby enhancing the induction of apoptosis in HeLa cells. 

*Apoptosis/Necrosis Assessment Using Annexin V/PI*

**Figure 4.**
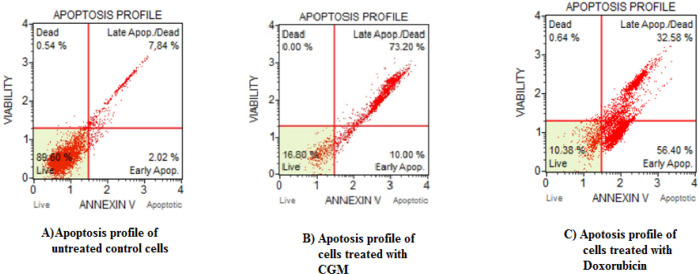
Apoptosis/Necrosis Assessment Using Annexin V/PI. The flow cytometry analysis of HeLA cells treated with 25 µg/ml concentration of CGM. The scatter plots show early apoptotic, late apoptotic, and necrotic cells following 48 h treatment. The values are expressed as Mean ± SD for 3 different experiments

Double staining by Annexin V-FITC and PI was performed to examine the apoptotic potential of CGM. The results obtained from the induction of apoptosis/necrosis using flow cytometry are summarized in Figure 4. The data obtained from flow cytometry indicates that CGM significantly induced cell death in HeLa cancer cells. After HeLa cells were treated with 25µg/ml concentration of CGM for 48 h, cells were stained with Annexin V- FITC and PI, which can distinguish between viable, early apoptotic, late apoptotic, and dead cell populations as presented in the lower left, lower right, upper right, and upper left of the quadrant of the density plot, respectively. Based on the Annexin V/ PI staining, 89.60% of HeLa control cells were found alive with values of 2.02%, 7.84%, and 0.54% of cells, which are a normal process for cells growing in cultures. The HeLa cells exposed to CGM significantly increased the late apoptotic and necrotic cells as compared with untreated control cells. An increase in the percentage of apoptotic and necrotic cells was found with the values of 73.20% at 25 μg/ml of CGM treatment (Figure 4). At 25 μg/ml concentration, CGM was found to induce apoptotic and necrotic cell death as compared with standard drug doxorubicin of 32.58%. Our results are in good accordance that CGM showed apoptosis/cell death due to its anti-cancer properties and its increased bioavailability effect. 

*Cell Cycle Analysis*

**Figure 5:**
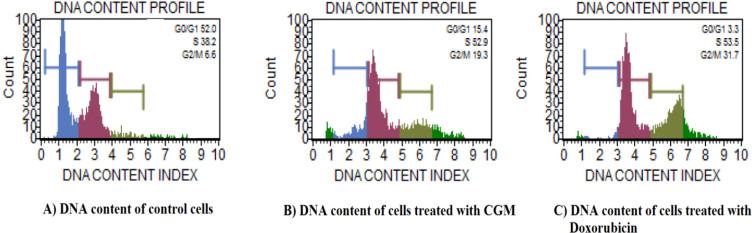
Cell Cycle Analysis. Cell cycle analysis in HeLa cells exposed to 25 µg/ml concentration of CGM for 48 h. Representative Flow cytometric image exhibiting changes in the progression of the cell cycle. Each histogram represents the percentage of cells arrested in different phases of the cell cycle. A) DNA content of control HeLa cells, B) DNA content of cells treated with CGM, C) DNA content of cells treated with standard drug doxorubicin

The results of cell cycle analysis in HeLa cell lines exposed to CGM at 25 μg/ml for 48 h are represented in Figure 5. The flow cytometric measurement of propidium iodide stained control and CGM-treated HeLa cells showed an increase in apoptotic S-phase. A significant increase in SubG1 and S-Phase arrest was observed at 25 μg/ml concentration of CGM -treated HeLA cells. The increase in the SubG1 and S-phase (apoptotic) population found in this study suggests that CGM treated HeLa cells were not able to go through the G2 checkpoint; therefore, G2/M transition was found to be affected. In the present study, cell cycle analysis demonstrated that CGM was able to arrest HeLa cells at Sub G1, S phase, which suggests that blocking S-phase cells might have associated with CGM induced apoptosis in HeLa cells. 

*Protein-Based Analysis by western blot*

**Figure 6:**
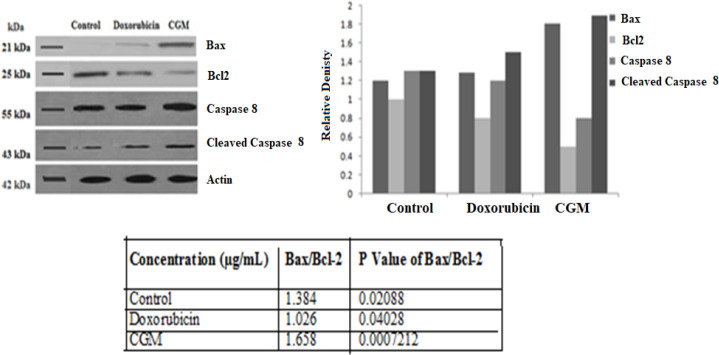
Protein-Based Analysis by western blot. Effect of CGM on Bax, Bcl-2, caspase- 8, and cleaved caspase-8 in HeLa cells using Western blot analysis. β -actin served as an internal control. The values are expressed as Mean ± SD for 3 different experiments

The underlying mechanism for the induction of apoptosis was assessed by protein-based Western blot analysis. By CGM treatment, the Bax and cleaved caspase-8 were upregulated, whereas Bcl-2 and the inactive form of caspase-8 were downregulated. From this data, it is confirmed that Bax, Bcl-2, cleaved caspase-8, and caspase-8 were involved in the apoptosis of HeLa cells induced by the CGM treatment (Figure 6). The present study demonstrates that the cytotoxic activity of the CGM extract on HeLa cells caused apoptosis induction in which may also be associated with both intrinsic and extrinsic pathways of cell death programme.

## Discussion

Nowadays, especially in developing countries, the spreading of cancer is dramatically increasing and thereby the rate of deaths is also too high. Therefore, scientists and researchers are giving more attention to alternative therapies and medicines to provide better treatment for cancer diseases owing to the fact that, most of the cancer therapies and medicines using for cancer patients have severe side-effects. In the present study, the results obtained from MTT and NRU assay showed that the CGM exhibited cytotoxic response at a low dose of 25μg/ml. The MTT and NRU assays are commonly used endpoints for cytotoxicity assessments, since they evaluate different aspects of cellular functions and, therefore, can be useful to examine the potential cytotoxic effects of CGM and CM. Estimation of cytotoxicity is generally based on uptake or exclusion of dye and are an indicator of the integrity of the plasma membrane or some intracellular organelles. However, the MTT assay indicates the mitochondrial function based on the enzymatic reduction of a tetrazolium salt by the mitochondrial dehydrogenase of viable cells (Mosmann, 1983). NRU is a measure of lysosomal integrity since it reflects the capacity of viable cells to incorporate vital dye into these organelles (Borenfreund and Puerner, 1987). Both MTT and NRU assays showed that CGM at 25μg/ml has a cytotoxic effect on HeLa cells as compared with CM. The morphological analysis reveals that HeLa cells were very sensitive to CGM, as in figure 2, especially at 25μg/ml concentration of CGM showed more cytotoxic as well as cell shrinkage and also the growths of HeLa cell were inhibited by CGM treatment. This result indicates that CGM significantly inhibited the proliferation of cervical cancer cells and induced apoptosis of cervical cancer cells.

ROS is highly responsible for the apoptotic activity as well as the key signaling molecules to modulate cell death (Wu and Bratton, 2013). ROS-mediated apoptotic signaling is mainly associated with mitochondrial dysfunction, the depletion of anti-oxidant status and loss of cellular redox balance. Several chemotherapeutic agents are exerting their anti-cancer efficacy via intracellular ROS generation. In the current study, CGM could induce ROS production in HeLa cells. CGM treated HeLa cells has high ROS generation which correlates with the mitochondrial membrane integrity, which might lead to a subsequent increase in apoptosis.

Evasion of apoptosis by cancer cells is a hallmark of cancer and induction of apoptosis by the cytotoxic anti-cancer agents is one of the approaches adopted for developing therapeutics for cancer treatment (Wong, 2011; Lowe and Lin, 2000). Based on morphological observation, we confirmed that CGM has the potency to induce apoptosis in HeLa cells. Therefore, we further investigated whether CGM induces apoptotic or necrotic cell death, by flow cytometry. It is well known that a high amount of ROS generation could lead to apoptotic and necrotic cell death (Foldbjerg et al.,2009). Excessive ROS generation has been linked with substantial DNA damage and apoptosis/necrosis (Hsin et al., 2008). From the findings, CGM at 25μg/ml exerted anti-cancer activity by inducing apoptosis. The cell cycle is a common and basic process for all living organisms. Based on the DNA synthesis, it is divided into G1-S-G2 phases. Therefore, targeting the cell cycle of cancer cells has emerged as an area of interest for cancer therapy. The apoptosis induction due to the presence of SubG1 peak in the process of cell cycle implies that the function of early and late apoptotic/necrotic pathway (Asharani et al., 2009; Ciftci et al., 2013). The results verified from cell cycle analysis showed that CGM can arrest HeLa cells at sub G1, S phase and hereby induced apoptosis. 

Apoptosis generally described as a mechanism in which cells die in response to their self-generated signals, is exemplified by various cellular changes such as loss of membrane integrity, chromatin condensation, mitochondrial swelling and DNA damage (Renault et al., 2015) and also an essential part in the cellular homeostasis as well as the treatment of cancer. Further, apoptosis is modulated by pro-apoptotic and anti-apoptotic proteins which are used as a target of anti-cancer therapy. Moreover, the mitochondria play important roles in apoptosis by controlling both pro-apoptotic and anti-apoptotic proteins. Therefore, apoptosis is mainly regulated by the action of two different pathways such as mitochondrial/intrinsic and death receptor/extrinsic pathway (Hongmei, 2012). In our study, we focused on the apoptosis-related protein in both pathways, namely, Bax and Bcl-2 (intrinsic pathway), and caspase-8 and cleaved caspase-8 (extrinsic pathway). Interestingly, in our study after 48 h of treatment, the Bax, a pro-apoptotic protein was increased, whereas the expression of Bcl-2 (anti-proapoptotic protein) and inactive caspase-8 continuously declined, together with a consecutive increase of active form cleaved caspase-8. Downregulation of the death suppressor Bcl-2 and activation of caspase-8 and bax might be inhibiting tumour growth and progression through promoting apoptosis. Moreover, apoptosis induction by intrinsic pathway is correlated with acceleration in Bax protein expression which can induce the release of cytochrome c from mitochondria and form an apoptosome complex. This complex can cleave inactive caspase-9 to an active form and then cleave the other effector caspases such as caspase-3 and caspase-7 leading to cell apoptosis. Meanwhile, the extrinsic pathway cleaved caspase-8, which is a key molecule that acts as initiator caspase that can lead to apoptosis condition. In addition, cleaved caspase-8 can cleave and translocate to mitochondria, where it further causes cytochrome c release, resulting in apoptosome formation, caspase activation and cell death (Beaudouin et al., 2013). In this study, treatment with CGM in HeLa cells showed upregulated Bax and cleaved caspase-8, whereas Bcl-2 and the inactive form of caspase-8 were downregulated. Therefore, it is confirmed that Bax, Bcl-2, cleaved caspase-8, and caspase-8 are involved in the apoptosis of HeLa cells induced by the CGM treatment. 

Taken together, our results lead us to suggest that the Curcumin galactomannoside complex showed high cytotoxicity on cervical cancer cells. Due to this cytotoxic effect, CGM exerts inhibition in cell growth and also in the core mechanism of action on the molecular level of apoptosis were seen in apoptosis and cell cycle arrest taking place at subG1/S -Phase in HeLa cells. It is to be concluded that the enhanced bioavailable curcumin galactomannoside (CGM) obtained from a food-grade formulation of natural curcuminoids can be used as a potent anti-cancer agent.

## Author Contribution Statement

None.
